# Classification of and detection techniques for RNAi-induced effects in GM plants

**DOI:** 10.3389/fpls.2025.1535384

**Published:** 2025-03-07

**Authors:** Cecilia Diaz, Steve U. Ayobahan, Samson Simon, Luise Zühl, Andreas Schiermeyer, Elke Eilebrecht, Sebastian Eilebrecht

**Affiliations:** ^1^ Department Ecotoxicology, Fraunhofer Institute for Molecular Biology and Applied Ecology IME, Schmallenberg, Germany; ^2^ Department Ecotoxicogenomics, Fraunhofer Institute for Molecular Biology and Applied Ecology IME, Schmallenberg, Germany; ^3^ Division I 3.2 Synthetic Biology Assessment, Enforcement of Genetic Engineering Act, Federal Agency for Nature Conservation (BfN), Bonn, Germany; ^4^ Department Plant Sciences & Bio-Hybrids, Fraunhofer Institute for Molecular Biology and Applied Ecology IME, Aachen, Germany

**Keywords:** RNAi GM plants, detection techniques, RNAi mechanism, off-target effects, RNAi pest control

## Abstract

RNA interference (RNAi) is a biotechnological tool used for gene silencing in plants, with both endogenous and exogenous applications. Endogenous approaches, such as host-induced gene silencing (HIGS), involve genetically modified (GM) plants, while exogenous methods include spray-induced gene silencing (SIGS). The RNAi mechanism hinges on the introduction of double-stranded RNA (dsRNA), which is processed into short interfering RNAs (siRNAs) that degrade specific messenger RNAs (mRNAs). However, unintended effects on non-target organisms and GM plants are a concern due to sequence homologies or siRNA-induced epigenetic changes. Regulatory bodies such as the EPA and EFSA emphasize the need for comprehensive risk assessments. Detecting unintended effects is complex, often relying on bioinformatic tools and untargeted analyses like transcriptomics and metabolomics, though these methods require extensive genomic data. This review aims to classify mechanisms of RNAi effects induced by short interfering RNA from different sources in plants and to identify technologies that can be used to detect these effects. In addition, practical case studies are summarized and discussed in which previously unintended RNAi effects in genetically modified plants have been investigated. Current literature is limited but suggests RNAi is relatively specific, with few unintended effects observed in GM crops. However, further studies are needed to fully understand and mitigate potential risks, particularly those related to transcriptional gene silencing (TGS) mechanisms, which are less predictable than post-transcriptional gene silencing (PTGS). Particularly the application of untargeted approaches such as small RNA sequencing and transcriptomics is recommended for thorough and comprehensive risk assessments.

## Introduction

RNA interference (RNAi) represents a cutting-edge approach in biotechnology for gene expression silencing, applied e.g. in plant protection, leveraging molecular principles to control gene expression. This innovative strategy encompasses both endogenous and exogenous applications, each with distinct methodologies and implications. Genetically modified (GM) plants harness RNAi to target plant endogenous transcripts e.g. to regulate the gibberellin pathway (maize event MON 94804) or to alter the fatty acid profile (soy event MON 87705). Endogenous applications also involve GM plants in a process known as host-induced gene silencing (HIGS) (Nowara et al., 2010) for pesticidal applications (e.g. maize event MON 87411 containing dsSnf7 against *Diabrotica*), (see https://euginius.eu). Conversely, exogenous applications, such as spray-induced gene silencing (SIGS), or root soaking of RNAi involve the direct application of RNA molecules to plants (Liu et al., 2020; Werner et al., 2020).

The core mechanism of RNAi in biotechnology application, such as plant protection, lies in its ability to selectively reduce the expression of specific genes within the target organism (Koeppe et al., 2023). In the majority of cases, this is achieved through the introduction of double-stranded RNA (dsRNA), which is subsequently processed by the RNase III Dicer or related enzymes to short interfering (si)RNA, whose base pairing with the complementary sequence of the target messenger (m)RNA leads to its degradation (Guo et al., 2016; Hung and Slotkin, 2021). While this sequence-based mechanism is advantageous for targeting pests and pathogens, there is a potential for unintended effects on non-target organisms (NTOs) and the GM plant itself (Christiaens et al., 2018). These effects may arise due to sequence homologies between the dsRNA and non-target mRNAs or through mechanisms such as siRNA-induced epigenetic changes and disruption of the organism’s endogenous RNAi pathways (Kloc et al., 2008; Zaratiegui and Martienssen, 2012; Swevers et al., 2013).

Recognizing the novel challenges posed by RNAi-based plant protection, regulatory bodies such as the US Environmental Protection Agency (EPA) and the European Food Safety Authority (EFSA) have acknowledged the need for comprehensive risk assessments (Christiaens et al., 2018; Papadopoulou et al., 2020; Christiaens et al., 2022). The Chemicals Committee and the Working Party on Chemicals, Pesticides and Biotechnology of the Organisation for Economic Co-operation and Development (OECD) have compiled considerations to integrate the latest scientific understanding into the environmental risk assessment of RNAi applications (Organisation for Economic Co-operation and Development (OECD) 2020).

One significant concern is the potential for unintended effects on GM plants themselves. Detecting these effects is complex due to several factors. Current prediction methods primarily rely on bioinformatic searches for complementary sequences to the siRNA within the GM plant’s transcriptome (Good et al., 2016; Lück et al., 2019; Farooq et al., 2021). However, these analyses are often hampered by the lack of a complete and accurate reference genome for the GM plant. When available, reference genomes of closely related cultivars may be used, but these can lead to inaccuracies due to sequence polymorphisms, resulting in false positives or negatives in off-target effect predictions.

In this review, we summarize the mechanisms by which RNAi applications could induce unintended effects in plants and evaluate the technologies and approaches available to detect these effects. By assessing the relevance of RNAi-mediated cellular mechanisms to GM plants based on existing literature, we provide a comprehensive overview and aim to rank these mechanisms according to their significance. This detailed examination will contribute to a better understanding of RNAi applications and the development of more accurate risk assessment methodologies.

## Mechanisms of RNAi-induced effects in plants

**Figure 1:**
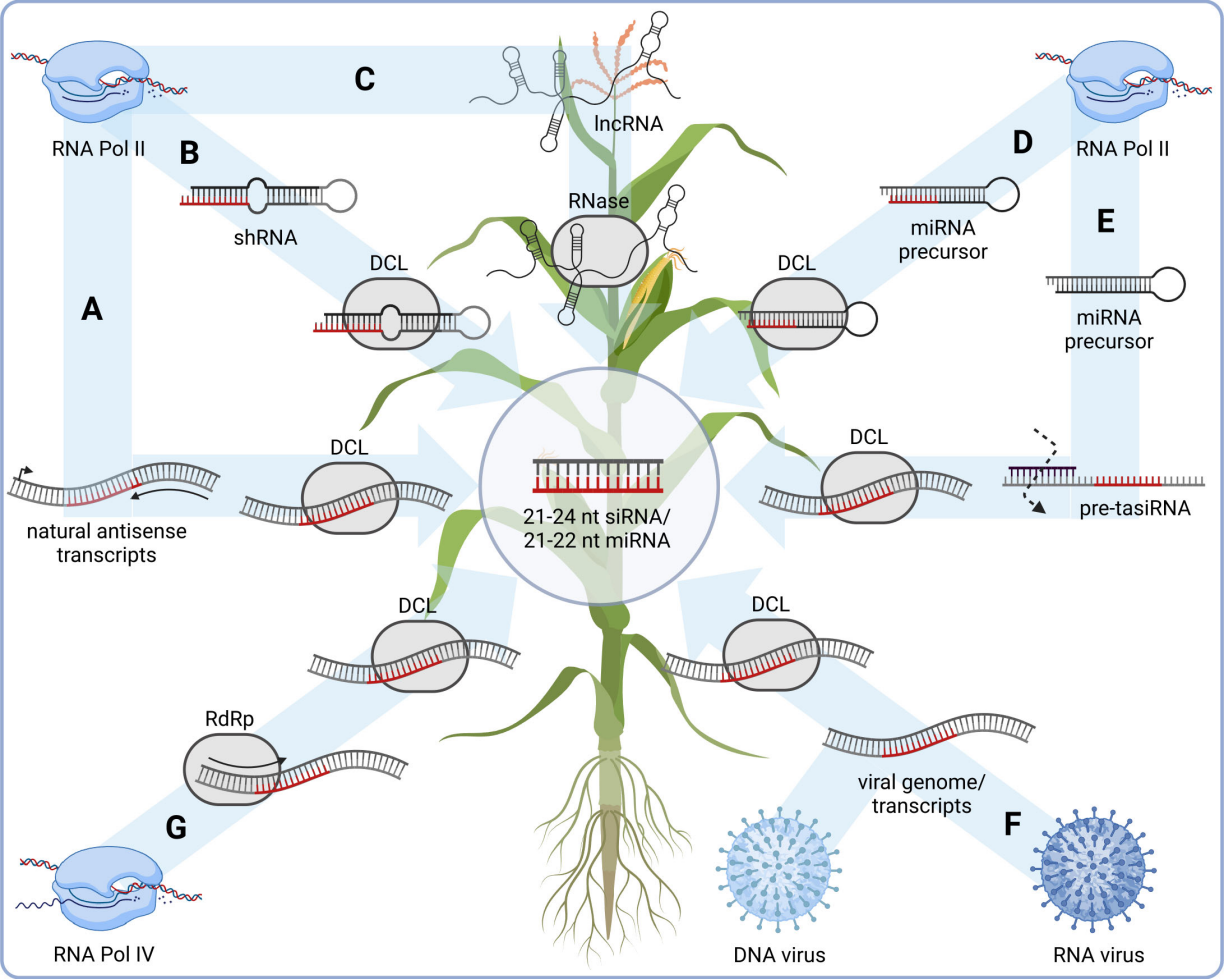
siRNA biogenesis in plants. **(A)** Synthesis of natural antisense transcripts by RNA polymerase II (RNA Pol II) followed by Dicer-like protein (DCL)-mediated cleavage. **(B)** RNA Pol II-mediated transcription of short hairpin (sh) RNAs, followed by DCL processing. **(C)** RNA Pol II-mediated synthesis of long non-coding (lnc) RNAs, followed by RNase digestion. **(D)** miRNA processing of RNA Pol II-transcribed miRNA precursors. **(E)** Trans acting (ta) siRNA pathway followed by siRNA synthesis by DCL. **(F)** Virus-derived siRNA synthesis from RNA or DNA viruses via replication/transcription followed by DCL processing. **(G)** RNA Pol IV-mediated transcription of double-stranded (ds) RNA as precursors for heterochromatic (hc) siRNA, followed by processing via DCL. Generated by the use of Biorender.com.

The principle of RNAi in plant protection relies on reducing or silencing the expression of specific essential genes in the target organism or the GM plant itself. These target genes typically belong to vital metabolic or developmental pathways, leading to a loss-of-function phenotype (Werner et al., 2020; Hernández-Soto and Chacón-Cerdas, 2021). RNAi-based pest control strategies primarily utilize two types of RNA precursors: short hairpin RNAs (shRNA), which consist of two complementary strands forming a stem-loop structure, and complementary dsRNA. The enzyme Dicer, found in nearly all eukaryotes with various isotypes (Zapletal et al., 2023), processes these precursor molecules into short, mostly 21-24 nucleotide (nt) RNA duplexes in the cytoplasm (**Figure 1**). In plants, Dicer-like (DCL) proteins play an important role in processing dsRNA into siRNAs of different length (Henderson et al., 2006; Mukherjee et al., 2013). The RNA duplexes include a guide strand and a passenger strand [reviewed in (Kim et al., 2009; Borges and Martienssen, 2015)]. While the passenger strand is degraded during further processing, the guide strand, which is complementary to the target gene sequence, is crucial for the silencing of the gene. In the following, we will first focus on the biogenesis of small RNAs in plants and then discuss the mechanisms of RNAi-based silencing before we discuss the implications of these mechanisms for possible off-target effects in GM plants.

### Biogenesis of small RNAs in plants

To investigate the effects of genetic modifications on the RNAi pathway in GM plants, it is essential to consider the natural mechanisms by which RNAi can affect gene expression in plants. To this end, the cellular pathways by which siRNA molecules can be produced in plants are first described here (**Figure 1**; **Table 1**). Precursors of siRNA are almost without exception double-stranded RNA molecules, which are either synthesized by endogenous RNA polymerases (RNA Pol) or introduced exogenously (Vazquez et al., 2004; Allen et al., 2005; Axtell et al., 2006). Endogenous precursors include natural antisense transcripts (NAT) synthesized by RNA Pol II, which base-pair with the sense mRNA of the coding gene and thus form the double-stranded substrate for corresponding RNases (**Figure 1A**) (Borsani et al., 2005; Jen et al., 2005; Zhang et al., 2012). RNA Pol II also synthesizes shRNAs encoded in the genome, which can then be processed by Dicer into siRNA (**Figure 1B**) (Wesley et al., 2001; Helliwell and Waterhouse, 2003; Senthil-Kumar and Mysore, 2011) or long non-coding (lnc) RNAs (Kim and Sung, 2012; Liu et al., 2012; Wu et al., 2012), whose secondary structures can have hairpins and can thus also be converted into siRNA by corresponding RNases (**Figure 1C**). Endogenously encoded micro (mi)RNAs are synthesized by Dicer or DCL1 in plants (Kurihara and Watanabe, 2004) from shRNAs, the miRNA precursors (**Figure 1D**), and either directly regulate the expression of target genes (by miRNA) or base-pair with the precursors of so-called trans-acting (ta)siRNAs, which are then generated by DCL from a double-stranded template (**Figure 1E**). Exogenously introduced precursors of siRNA are molecules introduced into a cell from an external source. A natural example are viral RNAs, which are either immediately present after infection and replication (RNA viruses, in plant viruses often single-stranded (ss) RNA genome) or are generated by transcription of the viral genome (DNA viruses) and are then templates for DCLs, which produce siRNA from them (**Figure 1F**) (Ruiz et al., 1998; Lu et al., 2003; Burch-Smith et al., 2004). RNA Pol IV or V can also be involved in siRNA synthesis, for example in the case of the synthesis of precursors of heterochromatic (hc)siRNAs, which are then converted to siRNA by DCL3 (**Figure 1G**) (Law and Jacobsen, 2010; Zhang and Zhu, 2011; Matzke and Mosher, 2014). In plants, the proteins DCL2, DCL3 and DCL4 generate siRNAs of different lengths mostly with 22 nt, 24 nt and 21 nt, respectively, which in turn trigger different mechanisms of silencing (Henderson et al., 2006; Mukherjee et al., 2013). Of note, DCL2-derived 22 nt siRNAs in plants are involved in a transitive and systemic spread of siRNA especially for antiviral defense, called secondary RNAi (Bouché et al., 2006; Chen et al., 2010; Garcia-Ruiz et al., 2010; Qin et al., 2017). This spread of RNAi involves the amplification and expansion of silencing signals that are mediated by RNA-dependent RNA polymerases (RdRp) (Sanan-Mishra et al., 2021). In this process, siRNAs act on longer RNAs (such as mRNA) as primers for RdRp, whereby a new, long dsRNA is synthesized, which is then eventually processed again by the RNAi machinery into siRNA triggering secondary RNAi.

**Table 1:** Mechanisms by which RNA interference induces gene expression changes in plants, categorized by their general mode of action, including the mechanism, the source of siRNA and corresponding references.

Category	Mechanism	Description	Source of siRNA	References
Post-Transcriptional Gene Silencing (PTGS)	siRNA-Mediated mRNA Degradation	siRNAs guide RISC to complementary mRNA, leading to its cleavage and degradation.	Trans-Acting siRNAs (ta-siRNAs)	(Vazquez et al., 2004; Allen et al., 2005; Axtell et al., 2006)
			Natural Antisense Transcripts (NATs)	(Borsani et al., 2005; Jen et al., 2005; Zhang et al., 2012)
			Exogenous short hairpin RNA (shRNA)	(Wesley et al., 2001; Helliwell and Waterhouse, 2003; Senthil-Kumar and Mysore, 2011)
			Long Non-Coding RNAs (lncRNAs)	(Kim and Sung, 2012; Liu et al., 2012; Wu et al., 2012)
			Virus-derived siRNAs	(Ruiz et al., 1998; Lu et al., 2003; Burch-Smith et al., 2004)
			Heterochromatic siRNAs (hc-siRNAs)	(Law and Jacobsen, 2010; Zhang and Zhu, 2011; Matzke and Mosher, 2014)
	siRNA-Mediated Translation inhibition	siRNAs guide RISC to complementary mRNA, leading to translation inhibition	Trans-Acting siRNAs (ta-siRNAs)	(Vazquez et al., 2004; Allen et al., 2005; Axtell et al., 2006)
			Natural Antisense Transcripts (NATs)	(Borsani et al., 2005; Jen et al., 2005; Zhang et al., 2012)
			Exogenous short hairpin RNA (shRNA)	(Wesley et al., 2001; Helliwell and Waterhouse, 2003; Senthil-Kumar and Mysore, 2011)
			Long Non-Coding RNAs (lncRNAs)	(Heo and Sung, 2011; Kim and Sung, 2012; Liu et al., 2012)
			Virus-Derived siRNAs (VIGS)	(Ruiz et al., 1998; Lu et al., 2003; Burch-Smith et al., 2004)
	miRNA-Mediated mRNA Degradation	miRNAs guide RISC to complementary or partially complementary mRNAs, resulting in cleavage or repression.	Endogenously expressed miRNAs	(Jones-Rhoades et al., 2006; Mallory and Vaucheret, 2006; Voinnet, 2009)
Transcriptional Gene Silencing (TGS)	DNA Methylation	siRNAs guide DNA methylation machinery to specific genomic regions, repressing transcription.	Long Non-Coding RNAs (lncRNAs)	(Wierzbicki et al., 2008; Zhang and Zhu, 2011; Matzke and Mosher, 2014)
			Heterochomatic siRNAs (hc-siRNAs)	(Law and Jacobsen, 2010; Zhang and Zhu, 2011; Movahedi et al., 2015)
			Pol IV/Pol V-derived siRNAs	(Law and Jacobsen, 2010; Zhang and Zhu, 2011; Matzke and Mosher, 2014)
			Endogenously expressed miRNAs	(Wu et al., 2010; Zhang and Zhu, 2011; Matzke and Mosher, 2014)
			Trans-Acting siRNAs (ta-siRNAs)	(Allen et al., 2005; Zhang and Zhu, 2011; Matzke and Mosher, 2014)
			Natural Antisense Transcripts (NATs)	(Borsani et al., 2005; Zhang and Zhu, 2011; Matzke and Mosher, 2014)
			Exogenous short hairpin RNA (shRNA)	(Waterhouse and Helliwell, 2003; Zhang and Zhu, 2011; Matzke and Mosher, 2014)
	Histone Modification	siRNAs direct histone-modifying enzymes to specific loci, causing chromatin condensation and gene silencing.	Long Non-Coding RNAs (lncRNAs)	(He et al., 2011; Zhang and Zhu, 2011; Liu et al., 2012)
			Heterochomatic siRNAs (hc-siRNAs)	(Law and Jacobsen, 2010; Zhang and Zhu, 2011; Matzke and Mosher, 2014)
			Pol IV/Pol V-derived siRNAs	(Law and Jacobsen, 2010; Zhang and Zhu, 2011; Matzke and Mosher, 2014)
			Trans-Acting siRNAs (ta-siRNAs)	(Axtell et al., 2006; Zhang and Zhu, 2011; Matzke and Mosher, 2014)
			Natural Antisense Transcripts (NATs)	(Borsani et al., 2005; Zhang and Zhu, 2011; Matzke and Mosher, 2014)
			Exogenous short hairpin RNA (shRNA)	(Helliwell and Waterhouse, 2003; Law and Jacobsen, 2010; Senthil-Kumar and Mysore, 2011)
			Virus-Derived siRNAs (VIGS)	(Baulcombe, 2004; Blevins et al., 2006; Matzke and Mosher, 2014)

### Mechanisms of RNAi-based silencing

**Figure 2:**
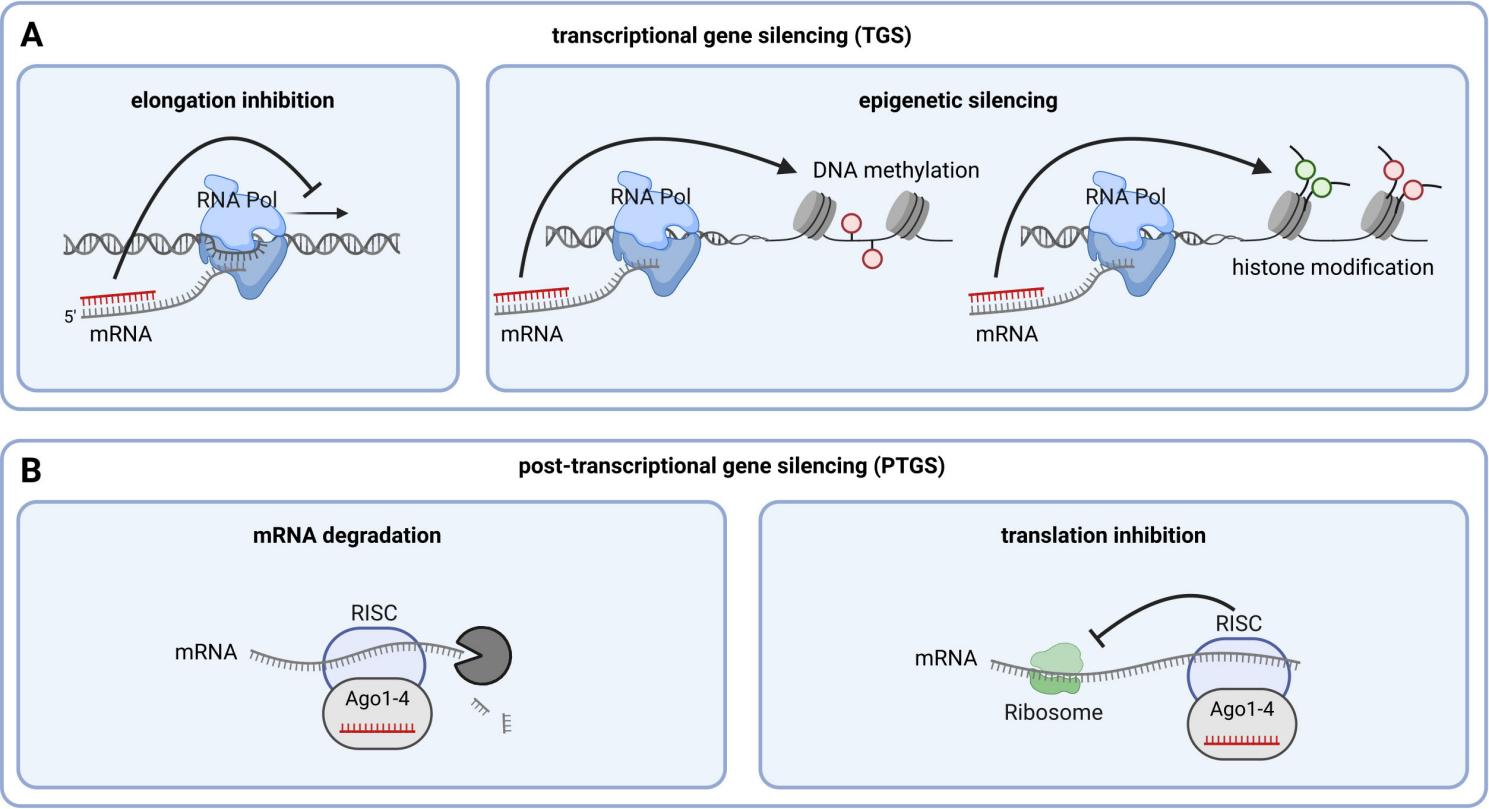
Mechanisms of RNAi-mediated silencing. **(A)** Mechanisms of transcriptional gene silencing (TGS). **(B)** Mechanisms of post-transcriptional gene silencing (PTGS). Generated by the use of Biorender.com.

Silencing mechanisms can occur in the GM plant harboring the RNAi construct, at the transcriptional level in the cell nucleus or the translational/post-transcriptional level in the cytoplasm (**Figure 2**). In the nucleus, siRNA can pair with the nascent mRNA of the target gene, recruiting factors to the transcription machinery that inhibit the transcription elongation by RNA polymerase (**Figure 2A**, left) (Guang et al., 2010). Similarly, siRNA can recruit enzymes that induce epigenetic silencing of the target gene through DNA methylation (Law and Jacobsen, 2010; Wu et al., 2010; Zhang and Zhu, 2011; Wu et al., 2012; Matzke and Mosher, 2014; Movahedi et al., 2015) or histone modification (Baulcombe, 2004; He et al., 2011; Liu et al., 2012) (**Figure 2A**, right) (Verdel et al., 2009). In plants, epigenetic silencing via DNA methylation is triggered by DCL3-generated ~24 nt siRNA involving a RISC complex containing the protein Argonaute (Ago)4 (Zilberman et al., 2003; Henderson et al., 2006; Qi et al., 2006; Zheng et al., 2007; Wierzbicki et al., 2009; Havecker et al., 2010; Olmedo-Monfil et al., 2010; Sarkies and Miska, 2014; Lewsey et al., 2016). The most well-studied RNAi silencing mechanism involves the degradation of the target gene’s mRNA (**Figure 2B**, left). In this process, the protein Ago recruits siRNA to the complementary mRNA sequence to form the RNA-induced silencing complex (RISC) (Wu et al., 2012). In plants, this is triggered by DCL4-generated ~21 nt siRNAs involving a RISC complex containing Ago1 (Xie et al., 2005; Qu et al., 2008; Chen et al., 2010; Wang et al., 2011). If there is perfect complementarity between siRNA and the target gene, the mRNA is degraded, leading to down-regulation of the target protein’s production (Valencia-Sanchez et al., 2006). With incomplete base pairing between siRNA and target mRNA, the RNA is not degraded; instead, ribosome-mediated translation is inhibited, resulting in reduced expression of the target gene (**Figure 2B**, right) (Brodersen et al., 2008).

The current literature suggests that siRNA molecules produced via different biogenesis pathways can differ in terms of their length, triggering different types of mechanisms of gene expression regulation described. While DCL4-generated 21 nt siRNA predominantly triggers PTGS via mRNA degradation, DCL3-generated 24 nt siRNA triggers TGS via epigenetic silencing and DCL2-generated 22 nt siRNA induces secondary siRNA. However, all DCL may act on long dsRNA molecules introduced into the plant. Therefore, both TGS and PTGS need to be considered when analyzing RNAi-induced effects in GM plants (**Table 1**).

### Implications of RNAi silencing mechanisms for possible off-target effects in GM plants

With regard to the knowledge about mechanisms by which RNAi can potentially induce unintended effects in the GM plant, the existing literature shows clear bias towards PTGS. For example, a PubMed search with the search term “RNAi AND PTGS NOT TGS” in title and abstract returned 118 hits, whereas the search term “RNAi AND TGS NOT PTGS” only returned 25 hits (as of 18.10.2024). Hence, most published studies are concerned with the investigation of effects resulting from the inhibition of translation or degradation of mRNA (possibly resulting from incomplete complementarity). Relatively fewer studies deal with TGS, possibly because here effects, for example via epigenetic silencing, could also arise upstream or downstream of the gene with sequence complementarity and these cannot be clearly determined on the basis of the pure small RNA sequence by analyzing complementary sequences in the genome.

## Techniques for the assessment of RNAi-induced effects in plants

RNAi can induce different types of off-target effects in the plant, which can be identified and studied using different techniques. Here we provide a brief overview of the different techniques that can be used to study the changes induced by RNAi and RNAi off-target effects. The methods employed to study RNAi effects can be divided into two main approaches: targeted and untargeted analysis (**Table 2**). Targeted screening of RNAi effects focuses on analyzing the intended silencing effects on specific target genes and includes, for example, validation of gene knockdown, functional assays, validation of phenotypic effects, assessment of specificity and long-term effects (e.g. stability of gene silencing). Targeted screening can also be used to analyze effects on predicted off-target genes. The corresponding techniques include molecular techniques such as RT qPCR (Chi et al., 2008; Sun and Rossi, 2009; Holmes et al., 2010; Varkonyi-Gasic and Hellens, 2011; Augustine et al., 2013; Kitzmann et al., 2013; Liu et al., 2014; Czarnecki et al., 2016; Keykha et al., 2016; Manske et al., 2017; Betti et al., 2021; Sarkar and Roy-Barman, 2021; Xu et al., 2021; Zhou et al., 2021; López-Márquez et al., 2023; Kyslík et al., 2024), northern blotting (Chi et al., 2008; Fukuhara et al., 2011; Augustine et al., 2013; Manske et al., 2017; Sarkar and Roy-Barman, 2021), western blotting (Kumar et al., 2003; Sahin et al., 2007; Sun and Rossi, 2009; Holmes et al., 2010; Liang et al., 2013; Han, 2018; Vidarsdottir et al., 2019; Kyslík et al., 2024), genetic techniques such as reporter gene assays (Kumar et al., 2003; Smart et al., 2005; Rinaldi et al., 2008; Sun and Rossi, 2009; Manske et al., 2017; López-Márquez et al., 2023) or genetic mutations (Chan et al., 2006; Czarnecki et al., 2016; Krzyszton and Kufel, 2022), phenotypic assays (Chi et al., 2008; Liu et al., 2014; Xu et al., 2021; Zhou et al., 2021; Tao et al., 2023), enzyme activity assays (Chi et al., 2008; Betti et al., 2021; Sarkar and Roy-Barman, 2021) or advanced techniques such as genome editing using CRISPR/Cas9 (Moore, 2015; Kanchiswamy et al., 2016; Peretz et al., 2018; Kleter, 2020; Mujtaba et al., 2021; Bock et al., 2022).

**Table 2:** Techniques for detecting RNAi off target effects in plants categorized by class and field. Short descriptions of each technique as well as the corresponding references are given.

Class	Category	Technique	Description	References
Targeted analysis	Molecular techniques	Quantitative RT-PCR (RT-qPCR)	Quantification of specific RNA molecules to assess reduction in target or off-target genes. advantages: high specificity; disadvantages: limited to a minimum RNA size, sequence information required, single gene analysis	(Chi et al., 2008; Sun and Rossi, 2009; Holmes et al., 2010; Varkonyi-Gasic and Hellens, 2011; Augustine et al., 2013; Kitzmann et al., 2013; Liu et al., 2014; Czarnecki et al., 2016; Keykha et al., 2016; Manske et al., 2017; Betti et al., 2021; Sarkar and Roy-Barman, 2021; Xu et al., 2021; Zhou et al., 2021; López-Márquez et al., 2023; Kyslík et al., 2024)
		Northern blotting	Quantification of specific RNA molecules to assess reduction in target or off-target genes as well as specific detection of siRNAs. advantages: high specificity, detection of fragments possible, detection of short RNA molecules; disadvantages: sequence information required, time-intensiveness, single gene analysis	(Chi et al., 2008; Fukuhara et al., 2011; Augustine et al., 2013; Manske et al., 2017; Sarkar and Roy-Barman, 2021)
		Western blotting	Quantification of specific proteins to assess reduction in target or off-target genes at the protein level. advantages: quantification of gene products, integrated assessment of mRNA degradation and translation inhibition; disadvantages: requires specific antibodies, single protein analysis	(Kumar et al., 2003; Sahin et al., 2007; Sun and Rossi, 2009; Holmes et al., 2010; Liang et al., 2013; Han, 2018; Vidarsdottir et al., 2019; Kyslík et al., 2024)
	Genetic techniques	Reporter gene assays	Assessment of the effect of RNAi on target gene expression in a GM reporter system. advantages: quick assessment, clear read out; disadvantages: artificial system, single gene analysis	(Kumar et al., 2003; Smart et al., 2005; Rinaldi et al., 2008; Sun and Rossi, 2009; Manske et al., 2017; López-Márquez et al., 2023)
		Genetic mutants	Comparison of RNAi effects with genetic mutants to validate phenotypic effects of gene knockdown. advantages: coverage of all phenotypic effects; disadvantages: mutant required, no discrimination between target and off-target effects	(Chan et al., 2006; Czarnecki et al., 2016; Krzyszton and Kufel, 2022)
	Phenotypic techniques	Phenotypic assays	Measurement of physiological parameters such as photosynthetic efficiency and hormone levels comparing RNAi-based GMP with unmodified comparator. advantages: identification of physiological parameters; disadvantages: no discrimination between target and off-target effects	(Chi et al., 2008; Liu et al., 2014; Xu et al., 2021; Zhou et al., 2021; Tao et al., 2023)
	Biochemical techniques	Enzyme activity assays	Measurement of activity of enzymes encoded by target genes to confirm functional consequences. advantages: assessment of functional consequences; disadvantages: restriction to the target gene, no assessment of off-target effects	(Chi et al., 2008; Betti et al., 2021; Sarkar and Roy-Barman, 2021)
	Advanced techniques	CRISPR/Cas9	CRISPR/Cas9 gene editing for validation of RNAi effects by knocking out target genes; Editing of potential off-target genes to assess phenotypic outcomes compared to RNAi treatments. advantages: specific analysis of phenotypic changes induced by target knockout; disadvantages: knowledge about off-target required, time consuming for a number of off-targets	(Moore, 2015; Kanchiswamy et al., 2016; Peretz et al., 2018; Kleter, 2020; Mujtaba et al., 2021; Bock et al., 2022)
Untargeted analysis	Molecular techniques	RNA sequencing (RNA-Seq)	Next generation sequencing of RNA and differential gene expression analysis to assess target and off-target genes. advantages: global detection of gene expression changes; disadvantages: time consuming, ideally availability of (high quality) reference genome required (workaround: *de novo* transcriptome assembly)	(Chan et al., 2006; Surget-Groba and Montoya-Burgos, 2010; Haque and Nishiguchi, 2011; Narzisi and Mishra, 2011; Jiao et al., 2021; Xu et al., 2021; Gaffo et al., 2022; Krzyszton and Kufel, 2022; Nguyen et al., 2022; Tyagi et al., 2022; Dong et al., 2023; López-Márquez et al., 2023; Budnick et al., 2024; Cazares et al., 2024)
		Small RNA sequencing (small RNA-Seq)	Next generation sequencing of small RNAs for siRNA and miRNA quantification e.g. to inform bioinformatic off-target predictions. advantages: global analysis of small RNAs; disadvantages: time consuming	(Huang et al., 2024)
	Phenotypic techniques	Visual observation	Observation of plant phenotypes such as growth rate, seed weight, leaf shape, and flower development. advantages: global assessment of phenotypic effects; disadvantages: no discrimination between target and off-target effects	(Augustine et al., 2013; Manske et al., 2017; López-Márquez et al., 2023)
		Microscopy	Microscopic observation cellular and subcellular changes. advantages: assessment of effects at the cellular and subcellular level; disadvantages: no discrimination between target and off-target effects	(Chi et al., 2008; Kitzmann et al., 2013; Betti et al., 2021; Sarkar and Roy-Barman, 2021; Xu et al., 2021; Zhou et al., 2021; Kyslík et al., 2024)
	Biochemical techniques	Metabolite profiling	Gas chromatography (GC) coupled mass spectrometry (MS) analysis of changes in metabolite levels to provide insights into affected metabolic pathways. advantages: global assessment of metabolites; disadvantages: time-consuming, no discrimination between target and off-target effects	(Chen et al., 2012; Huang et al., 2022; Baysoy et al., 2023; Bressan et al., 2023; Naik et al., 2023; Huang et al., 2024)
	Advanced techniques	Proteomics	Liquid chromatography coupled mass spectrometry (LC-MS) to identify changes in protein abundance and post-translational modifications. advantages: global detection of gene expression changes at the protein level; disadvantages: lower sensitivity, time consuming	(Chi et al., 2008; Lacourse et al., 2008; Asano and Nishiuchi, 2011; Chen et al., 2012; Naik et al., 2023)
		Chromatin immunoprecipitation sequencing (ChIP-Seq)	Assessment of changes in DNA methylation or histone modifications and transcription factor binding as a result of RNAi. advantages: global assessment of epigenetic TGS; disadvantages: time consuming	(Warnatz et al., 2011; Muhammad et al., 2020; Navarro-Mendoza et al., 2023)

Untargeted screening of RNAi effects involves comprehensive analyses mainly aimed at identifying unintended consequences and potential unpredicted off-target effects of RNAi treatments. These techniques include analyzing changes in transcriptomic profiles (Chan et al., 2006; Surget-Groba and Montoya-Burgos, 2010; Haque and Nishiguchi, 2011; Narzisi and Mishra, 2011; Jiao et al., 2021; Xu et al., 2021; Gaffo et al., 2022; Krzyszton and Kufel, 2022; Nguyen et al., 2022; Tyagi et al., 2022; Dong et al., 2023; López-Márquez et al., 2023; Budnick et al., 2024; Cazares et al., 2024), changes in protein expression (Chi et al., 2008; Lacourse et al., 2008; Asano and Nishiuchi, 2011; Chen et al., 2012; Naik et al., 2023) and modifications, metabolites (Chen et al., 2012; Huang et al., 2022; Baysoy et al., 2023; Bressan et al., 2023; Naik et al., 2023; Huang et al., 2024) and epigenetic changes (Warnatz et al., 2011; Muhammad et al., 2020; Navarro-Mendoza et al., 2023) to understand the downstream effects of RNAi on cellular processes. In addition, the distribution and potential off-target interactions of RNAi (small RNAs) with unintended mRNA targets can be determined. Furthermore, there are also bioinformatic tools that utilize computational algorithms to predict potential off-target sites based on sequence complementarity and thermodynamic stability (Good et al., 2016; Lück et al., 2019). However, such bioinformatic prediction tools require extensive knowledge, for example of the plant’s genome or its RNAi machinery, in order to apply them effectively.

When studying off-target effects of RNAi, both targeted and untargeted analyses offer unique advantages and disadvantages. Targeted analysis as focuses on predefined genes or pathways, provide specific and efficient validation of RNAi-induced gene silencing. It ensures detailed understanding of intended effects but has a limited scope, potentially missing broader biological impacts and introducing bias by overlooking unexpected interactions. These techniques require fewer technical resources and their costs are reduced, making targeted analysis well suited as validation techniques. In contrast, untargeted analysis provides a comprehensive, genome/proteome/transcriptome-wide assessment, enabling the discovery of both known and unknown off-target interactions. However, this approach depends on high-quality, well-annotated genomes for precise mapping of RNAi-induced changes and understanding the broader implications of gene silencing in plants. While this unbiased method generates extensive datasets that provide deeper insights into RNAi effects, it is resource-intensive and complex, demanding substantial time, computational power, and expertise for analysis and interpretation. Additionally, the large datasets can introduce noise, probably requiring further validation to identify meaningful effects. Despite potential challenges, combining both approaches can offer a balanced perspective, profiting the specificity of targeted analysis and the breadth of untargeted analysis to achieve thorough insights into RNAi effects.

## Discussion

### Relevance of unintended effects of RNAi for risk assessment

Unintended effects of RNAi applications in GM plants themselves are a critical focus in the safety assessment of food and feed. Consequently, the Food and Agriculture Organization of the United Nations, for example, has issued guidelines for conducting food safety assessments of food derived from recombinant DNA plants (Food and Agriculture Organization of the United Nations, 2003). Also the OECD publishes science-based consensus documents offering information for the regulatory assessments of specific food and feed products, including those derived from transgenic organisms (Organisation for Economic Co-operation and Development, 2021). These documents gather data on the product’s nutrients, anti-nutrients and toxicants, its use as food or feed, and other factors relevant to food and feed safety. Here and in various review articles on the topic of risk assessment of RNAi-based GM crops, primarily untargeted methods for analyzing gene products and their metabolites, such as proteomics and metabolomics, are proposed to investigate RNAi-induced effects in the GM crop itself (Senthil-Kumar and Mysore, 2011; Kleter, 2020; Papadopoulou et al., 2020; Chaudhary et al., 2024).

The mechanisms by which the RNAi pathway can trigger specific gene expression changes in plants include both transcriptional and post-transcriptional regulation. These processes rely on specific base pairing, either with the nascent transcript (TGS) or with the mature target mRNA or a sequence-like mRNA (PTGS). While 21 nt siRNAs are predominantly involved in PTGS, 24 nt siRNAs often trigger TGS via epigenetic changes. In PTGS the target gene is directly known based on the sequence, whereas TGS can also affect genes located in close or distant proximity to the gene with sequence homology, making sequence-based prediction of TGS induced effects more difficult. PTGS is by far the most investigated mechanism in scientific studies to date, while the literature on RNAi-induced TGS is relatively limited. Therefore, the sheer number of scientific studies and the focus on PTGS to date does not necessarily reflect the actual relevance of the respective mechanisms in the plant, making it difficult to rank them according to their potential for causing unintended effects in plants.

Scientific literature on case studies investigating unintended effects in RNAi-based GM crops is currently scarce. However, bioinformatic tools are being dynamically developed to predict intended target genes and potential unintended effects on off-target genes in the GM crop or NTOs in case of HIGS, leveraging sequence homology to enhance the accuracy and scope of these predictions (Chen et al., 2019). While these tools often reach their limits in NTOs due to the lack or deficient annotated-genomes, high quality annotations are available for model plants or major crops, enabling such tools to predict PTGS effects on plant off-target genes with a higher probability. However, there are also mechanisms (such as TGS) that are not based on direct sequence homology to the target and whose unintended effects cannot be easily predicted bioinformatically. In most cases, it can be assumed that off-target effects manifest themselves at the transcriptome level and can be measured using sufficiently sensitive methods.

### Adequate techniques to detect unintended RNAi-induced effects

To detect unintended RNAi-induced effects in GM plants for risk assessment, knowledge about the siRNAs processed in the GM plant, such as size and sequence, compared to the wild type is necessary. Since both intended and possible secondary siRNAs (such as tasiRNA) can play a role, untargeted analyses, such as small RNA sequencing, should be used to identify the sequences of all siRNAs. With this knowledge, bioinformatic tools can be used to predict both intended and unintended effects mediated by sequence homology, primarily through PTGS, and these predictions can be validated using targeted methods such as RT-qPCR. However, a comprehensive bioinformatic search for homologies requires access to the plant´s complete genome, whereas RT-qPCR analyses can also be managed with knowledge of shorter sequence segments. Unintended effects mediated by TGS, on the other hand, are not directly linked to the actual sequence of the siRNA and therefore cannot be adequately detected with targeted methods, but only with untargeted methods. RNA sequencing, for example, can be used for the direct, untargeted investigation of gene expression changes, changes in histone modifications can be detected using ChIP-Seq or altered DNA methylation patterns can be detected using bisulphite sequencing. However, all these methods require the availability of the plant´s genome for accurate analysis. Additionally, there are currently no studies that specifically address the importance of selecting appropriate plant material such as tissue type, developmental stage, and sampling time points or the sensitivities required for untargeted analyses to effectively capture RNAi-induced changes (e.g. alterations in gene expression). Most published studies have focused on using plant tissues, like leaves, without a detailed exploration on how these factors might influence the detection and interpretation of RNAi-induced effects. Likewise, unintended off-target genes may be expressed, for example, in certain tissue types and not in others. These gaps highlight the need for more comprehensive research to optimize experimental designs in RNAi studies aiming to identify unintended effects.

### Case studies assessing unintended effects

Among the few studies assessing unintended effects of RNAi in GM plants, some have employed untargeted omics methods to analyze changes in gene expression and metabolite profiles. For example, Huang et al. (2022) compared the leaves of three transgenic maize RNAi lines resistant to *Apolygus lucorum* with those of three conventionally bred maize lines. Using untargeted omics methods at the levels of small RNAs, the transcriptome and the metabolome, the authors observed that the number of differentially expressed genes (DEGs) and differentially accumulated metabolites (DAMs) were greater in RNAi lines than in conventional lines. Additionally, Zörb et al. (2013) using GC-MS-based metabolite profiling showed that RNAi-mediated silencing of the sulfur-rich alpha-gliadin storage protein family in wheat grains did not induce changes in any of the 109 metabolites analyzed. Similarly, Zhang et al. (2020) investigated transcriptomic and metabolomic changes in RNAi-based GM maize resistant to *Monolepta hieroglyphica* compared to its unmodified variant. This study only identified a single DEG at the transcriptome level and 8 out of 5787 metabolites as DAMs, leading the authors to conclude that the RNAi variant exhibited negligible changes compared to the wild type.

Building on the insights gained from studies exploring off-target effects in RNAi-based GM plants, these findings have helped to inform regulatory approaches, including the one of the first authorization-relevant risk assessments for an RNAi-based genetically modified crop was carried out by the US Environmental Protection Agency (US EPA) for SmartStax Pro (MON 87411/Unique ID: MON-87411-9) (EPA Reg. Number: 62719-707). As part of the product characterization and human risk assessment, in 2016 the US EPA recommended a number of methods to rule out unintended side effects. These include transcriptome analyses using microarray or RNA sequencing, proteome analyses, GC-MS-based metabolomics, and the global detection of changes in DNA methylation patterns. It should be noted that certain recommended methods, such as microarray analyses for transcriptome studies or 2D gel electrophoresis coupled with MS for transcriptome analysis, are no longer state-of-the-art and should be replaced by more up-to-date methods such as RNA sequencing and LC-coupled MS, respectively. The US EPA advised that these analyses should be carried out comparatively between the GM plant containing all modification events (SmartStax Pro), the GM plant lacking the dsRNA cassette (SmartStax) as well as non-genetically modified lines across several generations. Furthermore, they recommended using a combination of different omics methods and to combine them with more sensitive methods such as RT-qPCR, to thoroughly exclude unintended effects.

### Current limitations and future research

In summary, the challenges in detecting unintended RNAi effects in GM plants lie in the diversity of siRNAs that can be formed from corresponding precursor molecules and in the fact that TGS (especially via epigenetic mechanisms) can also affect the expression of nearby genes without sequence homology, indicating that targeted/biased bioinformatic methods alone are not sufficient for excluding unintended effects. The few available studies indicate that the RNAi method appears to be relatively specific with minimal unintended effects expected (Zörb et al., 2013; Zhang et al., 2020; Huang et al., 2024).

Untargeted approaches, such as RNA sequencing for transcriptome analysis, LC-MS-based proteomics or GC-MS-based metabolome profiling, offer a promising and increasingly sensitive means of investigating these effects. The current state of well-annotated plant genomes varies significantly across species, with high-quality annotations available for some model plants and major crops, while others remain underrepresented. This variability poses challenges for accurately mapping RNAi-induced changes, as comprehensive and well-annotated reference genomes are crucial for identifying both target and off-target effects, as well as for understanding the broader biological impact of RNAi in diverse plant species. One way around this problem is to perform a *de novo* assembly of the transcriptome of unannotated plants (Surget-Groba and Montoya-Burgos, 2010; Narzisi and Mishra, 2011). However, this depends on the quality and depth of the sequencing. In combination, bioinformatic approaches with untargeted methods, such as various omics, offer the possibility to detect specific off-target effects in GM plants.

Future research on detecting RNAi-induced effects in GM plants should focus on improving sensitivity and specificity with advanced sequencing technologies, better off-target detection through CRISPR, and more accurate quantification using methods like RT-qPCR and proteomics. Environmental impact studies, long-term monitoring, and standardizing protocols will be key for regulatory safety assessments.
